# Comparative Study between Laser Light Stereo-Lithography 3D-Printed and Traditionally Sintered Biphasic Calcium Phosphate Scaffolds by an Integrated Morphological, Morphometric and Mechanical Analysis

**DOI:** 10.3390/ijms20133118

**Published:** 2019-06-26

**Authors:** Carlo Mangano, Francesco Mangano, Luigi Gobbi, Oleg Admakin, Satoshi Iketani, Alessandra Giuliani

**Affiliations:** 1 Private Practice, 22015 Gravedona (CO), Italy; 2 Department of Materials, Environmental Sciences and Urban Planning, Polytechnic University of Marche, Via Brecce Bianche, 60131 Ancona, Italy; 3 Department of Prevention and Communal Dentistry, Sechenov First Moscow State Medical University, 119991 Moscow, Russia; 4 DWS S.r.l., 36016 Thiene (VI), Italy; 5 Department of Clinical Science, Polytechnic University of Marche, Via Brecce Bianche, 60131 Ancona, Italy

**Keywords:** laser light stereo-lithography (SLA), 3D-printing, biphasic calcium phosphate scaffolds, morphometric analysis, biomechanical analysis, *X*-ray microtomography

## Abstract

In dental districts, successful bone regeneration using biphasic calcium phosphate materials was recently explored. The present study aimed to perform a comparative study between 3D-printed scaffolds produced by laser light stereo-lithography (SLA) and traditionally sintered biphasic calcium phosphate scaffolds by an integrated morphological, morphometric and mechanical analysis. Methods: Biphasic calcium phosphate (30% HA/70% β-TCP) samples, produced by SLA-3D-printing or by traditional sintering methods, were tested. The experimental sequence included: (1) Microtomography (microCT) analyses, to serve as control-references for the 3D morphometric analysis; (2) loading tests in continuous mode, with compression up to fracture, to reconstruct their mechanical characteristics; and (3) microCT of the same samples after the loading tests, for the prediction of the morphometric changes induced by compressive loading of the selected materials. All the biomaterials were also studied by complementary scanning electron microscopy to evaluate fracture regions and surfaces. Results: The characterization of the 3D mineralized microarchitecture showed that the SLA-3D-printed biomaterials offer performances comparable to and in some cases better than the traditionally sintered ones, with higher mean thickness of struts and pores. Interestingly, the SLA-3D-printed samples had a higher ultimate strength than the sintered ones, with a smaller plastic region. Moreover, by SEM observation, it was observed that fractures in the SLA-3D-printed samples were localized in the structure nodes or on the external shells of the rods, while all the traditionally sintered samples revealed a ductile fracture surface. Conclusions: The reduction of the region of plastic deformation in the SLA-3D-printed samples with respect to traditionally sintered biomaterials is expected to positively influence, in vivo, the cell adhesion. Both microCT and SEM imaging revealed that the studied biomaterials exhibit a structure more similar to human jaw than the sintered biomaterials.

## 1. Introduction

Dental implantology is a valid and predictable practice to restore function and aesthetics in partially or completely edentulous patients; indeed, implants were shown to have long survival times, particularly in the mandible [1,2]. However, the correct placement of implants is achieved only when a sufficient alveolar bone volume is guaranteed [2,3,4].

Several pathologic processes, including absorption of alveolar bone after tooth loss (due to a lack of mechanical loading), periodontal diseases, traumatic injuries, cysts, and tumors, often produce severe alveolar bone defects, preventing the implants to be placed correctly [5]. In these cases, alveolar ridge augmentation is indicated [3,4,5].

Biomaterials can enhance the body’s natural ability to regenerate lost bone. Indeed, the newly forming bone needs a scaffold to drive and support the regeneration process. Several materials were used in dentistry and implantology as bone substitute biomaterials (BSBs) in order to promote and increase tissue regeneration. These BSBs are nowadays classified into four groups according to their origin: Autogenic (i.e., bone explanted from the same patient), allogeneic (i.e., bone explanted from another person), xenogenic (bone originating from animal models) and synthetic (with no biological origin).

Freezing or freeze-drying processes reduce the risk of an immune response of a bone graft after surgery, but sterilization itself weakens the mechanical property of a grafted bone up to approximately 50%. Gamma radiation adversely affects the mechanical and biological properties of bone allografts by degrading the collagen in bone matrix. These effects are dose-dependent and give rise to a dose-dependent decrease in mechanical properties of allograft bone when gamma dose is increased above 25 kGy for cortical bone or 60 kGy for cancellous bone [6]. The elastic limit and resilience were significantly increased by irradiation with the dose 25 kGy, but not 35 kGy, when the time of irradiation was longer. Additionally, irradiation at ambient temperature decreased maximum load, elastic limit, resilience, and ultimate stress [7].

Xenogenic and synthetic biomaterials were revealed as successful, being osteoconductive and not requiring to obtain bone from the patient by additional surgical procedure with possible concurrent increase in morbidity [8].

Micro- and macro-porous biphasic calcium phosphate, produced by a combination of hydroxyapatite (HA) and tricalcium phosphate (TCP), have been largely proposed and characterized in oral surgery procedures [9,10,11,12]. In vitro studies showed that biphasic calcium phosphate scaffolds were cytocompatible, also enhancing cell viability and proliferation, as compared with pure TCP [11]. Moreover, they can show two types of porosity: The microporosity, with presence of pores smaller than 10 µm, for the biological fluids moving and ion exchanging, and the macroporosity, with pores ranging from 300 to 600 µm, enabling the colonization by the osteogenic cells [8].

In recent years, several techniques have been tested to produce ceramic BSBs, including rapid prototyping. This method, by computer-aided design files, is able to plot a 3D scaffold, offering precise control of its geometry and structural properties.

Mangano et al. [13] reported the study of a scaffold, manufactured through the rapid prototyping technique that was used as graft for sinus augmentation in an animal model of sheep. They evaluated the occurrence and progression of the bone regeneration process and the scaffold resorption rate during a total healing time of 90 days. The same authors [14] also evaluated the bone regenerative property of the amniotic epithelial cells, loaded on the same 3D-printed scaffold, and grafted in an animal (sheep) model. Sinus explants showed in the grafted region a reduced fibrotic reaction, a limited inflammatory response and an accelerated process of angiogenesis. The same 3D-printed biphasic calcium phosphate ceramics were also tested in clinical studies by assessing histological and histomorphometric results of human specimens retrieved from sinuses augmented with these scaffolds [15]. Comparing them to anorganic bovine bone, mineralized solvent-dehydrated bone allograft and equine bone, after a healing period of six months, the results supported the successful use of HA/TCP BSBs for sinus augmentation. The same type of scaffold, produced by CAD/CAM procedures, was also used for revealing the alveolar ridge augmentation six months [16] and one year [17] after grafting, showing compact mature bone remodeling, marrow spaces, and newly formed trabecular bone surrounded by residual BSB particles. Moreover, other clinical studies demonstrated the high biocompatibility and osteoconductivity of HA-beta-TCP 30/70 for sinus augmentation procedures [10,18].

The 3D-printing method introduced in the present study is the laser light stereo-lithography (SLA), an innovative process for printing bioceramics. To the authors’ best knowledge, there are few studies in the literature on this method. In particular, Carrell et al. [19] investigated the performance of these scaffolds to promote the vertical growth of cortical bone in a sheep calvarial model. They obtained a porous structure, highly osteoconductive, producing a bone mass that, two months after the scaffold grafting, was four times greater than that obtained with standard substitutes. However, as reported by Bose et al. [20], despite the growing interest in the SLA technique due to its ability to perform complex internal features (which facilitate in-vivo patterning of growth factors, proteins and cells), at present the method is applicable only to photopolymers, with limited production testing. Thus, the investigation of SLA-produced scaffolds is still in progress and includes the testing of material composition, architecture, structural mechanics, surface properties and properties of degradation products, together with the composition analysis of any added biological component and, of course, the study of the changes of all these factors with time [21].

In this context, different imaging techniques were applied to investigate the properties of biomaterials proposed to act as scaffolds. In particular, allowing an accurate 3D examination, high-resolution tomography (microCT) was not only employed to reconstruct the complex architecture of bone tissue at different scales [22,23,24,25] and in different genetic and environmental conditions [26,27,28,29], but it is also increasingly became a powerful tool for the characterization of biomaterials and engineered bone in the different skeletal sites. Indeed, interesting microCT studies have been performed on different biomaterials that have previously been indicated as bone-substitute [30].

In dental districts, successful bone regeneration using biphasic calcium phosphate materials was recently explored using microCT: Special 3D morphologies, in the shape of granules or structured blocks, were tested either in an acellular strategy (pure scaffold grafting and its colonization by endogenous cells) [9] or combining the biomaterial with cells in vitro [14]. A recent clinical study [10] reported a quantitative kinetics evaluation of blocks versus granules in biphasic calcium phosphate scaffolds. While the morphometric parameters were comparable up to five or six months from grafting, nine months after grafting, microCT revealed that the sites grafted with blocks mimicked the healthy native bone of the maxillary site slightly better than those grafted with particulates, also guaranteeing in the meantime a better biomechanical stability.

Indeed, mechanical testing of these biomaterials can be another critical step in both their design and next clinical evaluation. Their mechanical integrity is a critical design consideration, and retention of mechanical properties after in vivo grafting can serve as an indicator of the biocompatibility. Conversely, the decline in mechanical properties of these biomaterials can contribute to the understanding of temporal and spatial characteristics of the degradation process inside the hosting jawbone, with the final goal to evaluate their efficacy and/or safety after in vivo grafting.

The biomaterial strength describes its general integrity; it has to be matched and compared to the strength of the jawbone, which is complex and presents multiple structural levels and an array of biomechanical properties. Key biomechanical properties of bone include stiffness, toughness, ductility and mechanical strength. When measured in bone tissue, these properties are known as the intrinsic biomechanical properties of bone, while the extrinsic biomechanical properties reflect the structural behavior of the whole jaw [31].

In the present study, SLA-3D-printed biphasic calcium phosphate scaffolds were studied, for the first time to the authors’ knowledge, by combining their morphological and morphometric 3D analysis with an innovative study of their mechanical properties, before and after compressive tests in continuous mode up to fracture.

## 2. Results

All the biomaterials presented a biphasic composition: 30% of hydroxyapatite (HA) and 70% beta tricalcium phosphate (β-TCP). The balancing of the two phases was chosen in order to preserve, during grafting, the overall bone volume, guaranteed by the HA low resorption, and to achieve, in the meantime, the target bone regeneration favored by the β-TCP rapid reabsorption [10,16].

The analyses were performed using a microCT SkyScan-Bruker 1174 laboratory system, completed with a Material Testing Stage (MTS1) and a SEM device, all available at the CISMIN Centre of the Polytechnic University of Marche (Ancona, Italy). The overall study was focused on the following objectives:

The morphological and morphometric analysis of the internal porosity; indeed, an adequate porosity allows cell migration, proliferation and activity after grafting, therefore achieving osteoconduction and, possibly, osteoinduction and neo-vascularization;

The analysis of biomaterials’ resistance to compressive forces. Indeed, the implant insertion inevitably produces compressive forces that cause an alteration of the biomaterial performance and of the overall jaw homeostasis: If the latter exceeds the resistance limit values, a relevant inhibition of the osteoblasts activity and consequent bone resorption may occur.

### 2.1. MicroCT Analysis

The microCT analysis was focused on synthetic SLA-3D-printed samples, candidates for use as bone substitutes in jaw sites as possible alternatives to traditionally sintered biomaterials of the same composition.

**Figure 1:**
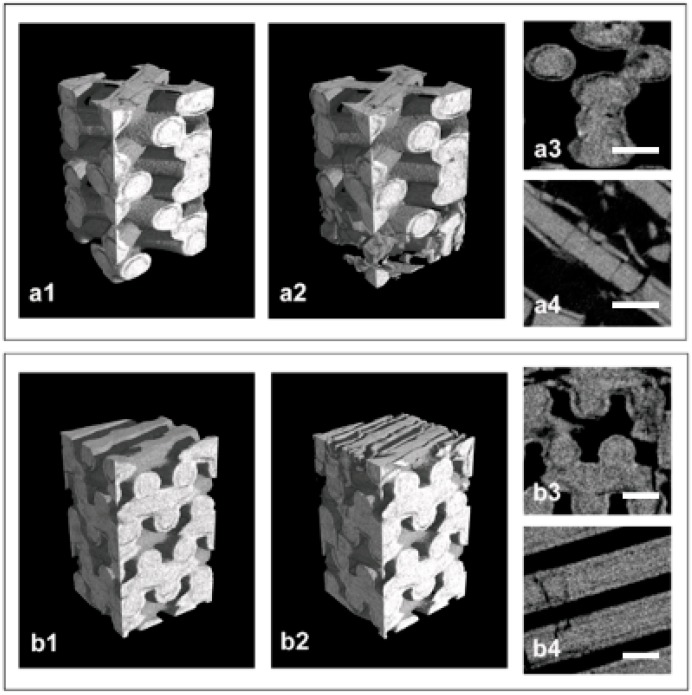
Microtomography (MicroCT) analysis of the laser light stereo-lithography (SLA)-3D-printed biomaterials: (**a1–a4**) Printed@BigPores sample; (**b1–b4**) Printed@SmallPores sample—3D reconstruction before (**a1**,**b1**) and after (**a2**,**b2**) compressive loading, and axial (**a3**,**b3**) and sagittal (**a4**,**b4**) 2D sections after fracture. A slight inhomogeneity in distribution of the biomaterial inside the rods is clearly visible in both sample types, resulting in an abnormal porosity that recurs throughout the sample in the layers closest to the surface of the rods. In the 2D sections, it is clearly shown that these porosities are responsible for the fracture of the sample after compressive loading. Scale bars: 400 µm.

MicroCT images of representative SLA-3D-printed samples are reported in Figure 1, in particular the 3D microCT reconstruction of a Printed@BigPores sample (group a) and of a Printed@SmallPores sample (group b), before (Figure 1a1,b1) and after (Figure 1a2,b2) compressive loading up to fracture, and a transversal (Figure 1a3,b3) and a longitudinal section (Figure 1a4,b4) of the same fractured samples.

**Figure 2:**
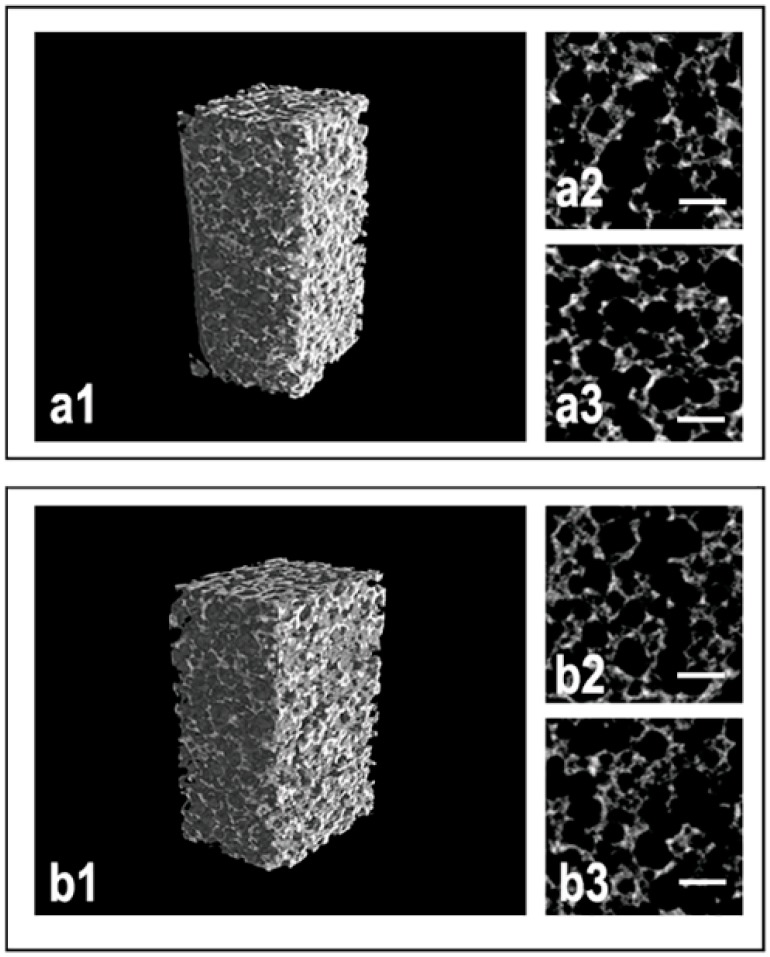
MicroCT analysis of the traditionally sintered biomaterials: (**a1–a3**) Sintered@*T* = 1150 °C sample; (**b1–b3**) Sintered@*T* = 1280 °C sample—3D reconstruction (**a1**,**b1**) after compressive loading, and axial (**a2**,**b2**) and sagittal (**a3**,**b3**) 2D sections after fracture. After the fracture, a decrease in the connectivity of the trabecular structures was shown, with evidence of discontinuity surfaces subsequently studied by electron microscopy. Scale bars: 400 µm.

MicroCT images of traditionally sintered samples are reported in Figure 2, in particular the 3D microCT reconstruction of a Sintered@*T* = 1150 °C sample (group a) and of a Sintered@*T* = 1280 °C sample (group b), after (Figure 2a1,b1) compressive loading up to fracture, and a transversal (Figure 2a2,b2) and a longitudinal section (Figure 2a3,b3) of the same fractured samples.

A morphometric analysis of these SLA-3D-printed samples was carried out, comparing Printed@BigPores with Printed@SmallPores samples, but also observing similarities and/or mismatches with sintered biomaterials of identical composition (Sintered@*T* = 1150 °C and Sintered@*T* = 1280 °C samples) and with healthy jawbone (bone control (ctr)). The quantitative descriptors are reported in Table 1.

The characterization of the 3D mineralized microarchitecture showed that the SLA-3D-printed biomaterials offer performances comparable to the traditionally sintered ones.

In general, the SLA-3D-printed samples revealed smaller sample surface to sample volume ratio (SS/SV) and mean strut number (S.Nr.) compared to the sintered samples and to the bone control.

For what concerns the sample volume to total volume ratio (SV/TV) parameter, the values found in the Printed@BigPores samples were comparable with those relative to the sintered samples and to the bone control. Conversely, the SV/TV of Printed@SmallPores samples were considerably higher than in the other groups and in bone control.

Consistently, significantly higher mean strut thickness (S.Th.) was observed in SLA-3D-printed samples both with respect to the sintered samples and with respect to bone control.

Interestingly, mean strut spacing (S.Sp.) was comparable with the same parameter in the bone control only in the Printed@SmallPores group.

After the compression test (ACT), reduced S.Th. and S.Sp. compared to before the test (BCT), due to the fragmentation of the structure caused by the fracture, were observed in all the groups.

### 2.2. Mechanical Characterization

The mechanical properties of the investigated biomaterials were studied during axial compressive tests, in continuous mode, up to fracture, using an MTS1 stage coupled to the Bruker SkyScan 1174 microCT device.

The engineering stress (σ) and engineering strain (ε) parameters were measured, where:

σ= F/A_0_ (1)

is the compressive force applied perpendicular to the average cross section of sample and:

ε = |(l_i_-l_0_)/l_0_| (2)

is defined, in the conventional way, by an l_0_ that is the length of sample before loading and by an l_i_ that is the length during testing.

The average cross section A_0_ of each sample was found, by microCT morphometric analysis, taking into account the real contact surface, i.e., subtracting any surface porosity.

**Figure 3:**
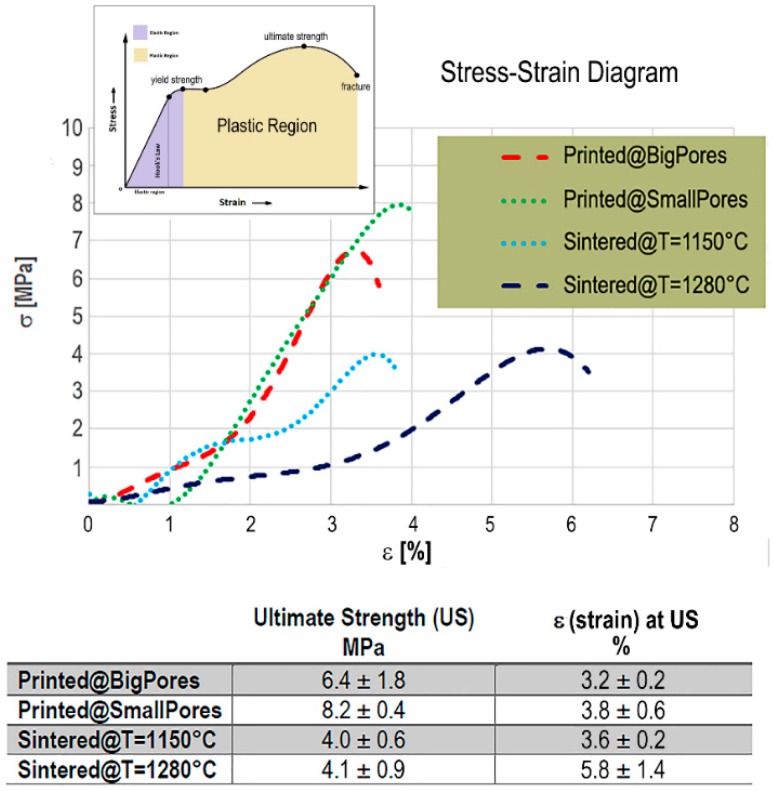
Top: stress (σ)–strain (ε) profiles of the investigated biomaterials. Bottom: Ultimate strength (mean ± SD, expressed in MPa) vs. ε (mean ± SD, expressed in %). The SLA-3D-printed samples with small pores (Printed@SmallPores samples) showed higher ultimate compressive strength with respect to SLA-3D-printed samples with big pores (Printed@BigPores samples). In the traditionally sintered samples, the strain ε at the yield point is around 1.4–1.6%, independent of the sintering temperature; however, despite a very similar ultimate strength (~4 MPa), the strain achieved there by the Sintered@*T* = 1280 °C samples was significantly larger than in Sintered@*T* = 1150 °C samples.

By applying Equations (1) and (2), the stress–strain profiles (σ vs ε) of the investigated biomaterials were obtained; they are shown in Figure 3. For the SLA-3D-printed biomaterials, the presence of small pores and thick rods (Printed@SmallPores samples) seemed to slightly improve the compressive strength with respect to samples with big pores (Printed@BigPores samples), even if the high variability of the latter group, as shown in the table in Figure 3, does not allow to establish certain information in this regard.

The SLA-3D-printed samples showed the typical stress–strain profile of brittle ceramics, with difficulties to measure the yield strength and showing a reduced region under plastic deformation, with special reference to the Printed@SmallPores group. However, in the case of the Printed@SmallPores samples, the presence of small pores and thick rods seems to increase the compressive ultimate strength of the biomaterial. In fact, while in Printed@BigPores samples an average compressive stress of 6.4 MPa was shown to be sufficient to achieve breakage after a compressive strain ε of about 3.6%, in Printed@SmallPores samples a compressive ultimate stress of approximately 8.2 MPa was shown necessary to achieve the fracture, after similar compressive strain (Figure 3).

On the contrary, the traditionally sintered samples, before ultimate strength and fracture, exhibited a yield point when ε ≅ 1.4–1.6%. This behavior was shown to be independent of the sintering temperature, which, in turn, seemed to influence only the ultimate strength. Indeed, it was observed that, against a similar ultimate stress of around 4 MPa, the strain achieved by the samples sintered at the higher temperature (Sintered@*T* = 1280 °C samples) was significantly larger (with ε ≅5.8%) than in samples sintered at lower temperature (Sintered@*T* = 1150 °C samples), where the strain ε, at the ultimate strength point, was ≅3.6%. This finding could be due to evidence described in the literature [32] for samples sintered at 1280 °C where different phases (alpha and others) were found that did not appear in the samples sintered at 1150 °C, in which only the desired phases were present.

Overall, the SLA-3D-printed samples had higher ultimate strength than the traditionally sintered ones, with a plastic region significantly smaller, possibly indicating a reduced energy absorption of the biomaterial under load up to fracture.

### 2.3. SEM Morphological Analysis

As a result of the minor toughness, revealed by the mechanical characterization under compressive loading, the fracture surfaces in the SLA-3D printed samples appeared at SEM very different from the fractures of the traditionally sintered samples.

**Figure 4:**
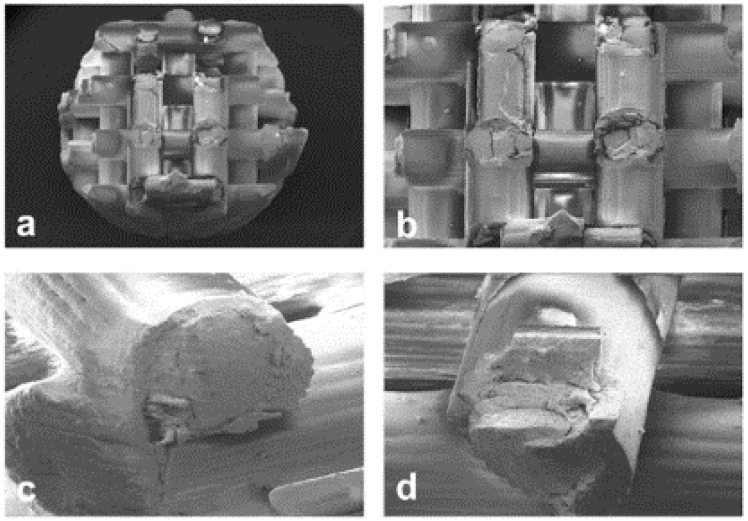
(**a**–**d**) Scanning electron microscopy images, at different original magnifications, of the Printed@BigPores samples after compressive loading up to fracture. (**c**,**d**) The rods are made of concentric cylindrical shells of biomaterial: The superficial shell, after fracture, seems to have some mismatches (porosity) with the inner shell, that is, most likely, the cause of the fracture under loading. Original magnification: (**a**) 16×; (**b**) 27×; (**c**,**d**) 77×.

SEM images, at different original magnifications, of a representative Printed@BigPores sample after compressive loading up to fracture are shown in Figure 4. These images confirm the microCT evidence, i.e., that the biphasic calcium phosphate rods presented cylindrical shells of biomaterial. The superficial shells, after fracture, exhibited some mismatches (porosity) with the inner one (Figure 4c,d), most likely inducing the fracture under loading.

**Figure 5:**
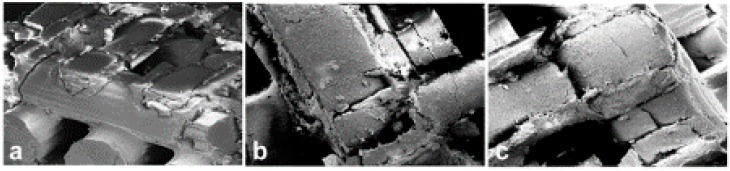
(**a**–**c**) Scanning electron microscopy images, with different details, of the 3D printed bone substitute biomaterials (BSBs) (small pores) after compression loading. The structure made of concentric cylindrical shells is present also in this case, with fractures originating from the exfoliation of these shells, especially in the nodes of the structure (**b**,**c**). Original magnification: (**a**) 46×; (**b**) 89×; (**c**) 90×.

Scanning electron microscopy images, with different details, of the Printed@SmallPores samples after compressive loading up to fracture are shown in Figure 5. Similar to Printed@BigPores samples, the structure of the rods is made of concentric cylindrical shells also in this case, with fractures originating from the exfoliation of these shells, especially in the nodes of the structure (Figure 5b,c).

**Figure 6:**
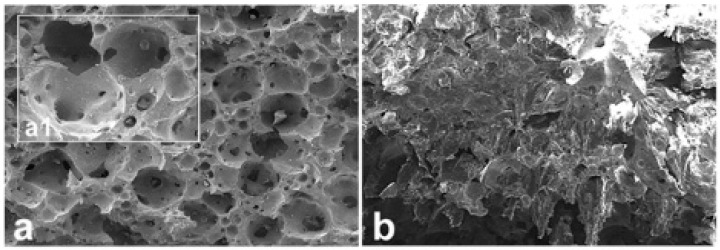
SEM micrographs of a representative sample of the Sintered@*T* = 1280 °C group. (**a**) Original microstructure: A network of well-connected pores with different dimensions. (inset **a1**) Same areas at higher original magnification; (**b**) Fracture surface: Exfoliation after compressive loading with damaged porous network. Original magnification: (**a**) 43×; (insert **a1**) 171×; (**b**) 26×.

In Figure 6, some SEM micrographs referred to Sintered@*T* = 1280 °C samples are reported. Similar images, but not shown, were obtained for the samples of the Sintered@*T* = 1150 °C group. In Figure 6a, the original microstructure was shown (at higher original magnification in the inset a1), indicating a network of well-connected pores with different dimensions. In Figure 6b, the fractured surface caused by compressive loading was shown: The porosity network is damaged, with surfaces exfoliation.

## 3. Discussion

Successful bone regeneration using biphasic calcium phosphate materials has been reported in the recent literature, including clinical applications in dental districts [33]. However, no experiences of integrated morphological, morphometric and mechanical analysis have been reported so far on these biomaterials.

In the present study, block-based biphasic calcium phosphate scaffolds, produced by SLA-3D-printing, were studied, for the first time to the authors’ knowledge, by an innovative integrated approach based on SEM morphological, microCT morphometric and mechanical analysis, before and after compressive tests up to fracture. The results were compared with those obtained on traditionally sintered biphasic calcium phosphate scaffolds with identical composition.

The characterization by microCT showed that the 3D mineralized microarchitecture of the SLA-3D-printed biomaterials seems to be comparable to, for some aspects better than, the microarchitecture of the traditionally sintered blocks.

Indeed, all the studied scaffolds closely mimicked the peculiar pores/struts architecture of the trabecular porous organization of the jawbone, characterized by relevant medullar spaces interconnectivity. The mean size (referred to in the S.Sp. index) of the macropores in the SLA-3D-printed biomaterials was found to be well above 100 μm, which is the size strongly recommended for bone tissue engineering scaffolds in order to achieve, in vivo, the osteointegration of the biomaterial [16,34], allowing bone cells migration and colonization into the biomaterial as well as its vascularisation [35].

Moreover, we have observed that the SLA-3D-printed samples revealed very small specific surface and number of struts compared to the traditionally sintered samples and to the bone control: This evidence could lead us to wrongly believe in a greater mechanical stability and resistance of the traditionally sintered samples compared to the SLA-3D-printed ones. Conversely, the significantly higher struts thickness observed in SLA-3D-printed samples with respect to the sintered samples seems to justify the higher ultimate strength we measured in the SLA-3D-printed biomaterials.

Furthermore, in the view of an integrated analysis by different methods, also mechanical properties of the SLA-3D-printed biomaterials have to be studied.

It has to be considered that the jawbone is daily submitted to several physical solicitations: Gravity force, body force, moments when speaking and eating and compression. During loading (stress action), such as during mastication, the jaw is submitted to strain. The graphical relationship between stress and strain is non-linear for the jawbone, due to yielding during compression caused by debonding of osteons at cement lines and microfractures [36]. Moreover, based on the testing of large specimens, the compressive strength of cancellous bone is normally included in the range of 2–12 MPa [37,38].

On the other hand, mechanical characterization of biomaterials that interface with bone tissues is fundamental to develop constructs with appropriate mechanical strength. Meanwhile, the mechanical properties of biphasic calcium phosphate biomaterials have been under investigation to design and to control their performance in jawbone grafting [39].

Thus, combined to the morphometric analysis, the study of the SLA-3D-printed BSB mechanical response to solicitations is also a key property to evaluate the contribution of the biomaterial to repair the jaw: Indeed, it should have mechanical properties comparable to those of the bone to be replaced.

In our study, the Printed@SmallPores group, i.e., the SLA-3D-printed samples with small pores and thick struts, appeared to be as good a candidate as BSB in terms of compressive mechanical performances, because it was shown to have higher strength to compression than the traditionally sintered biomaterials, with reduced regions under plastic deformation. Indeed, in order to remain injury free, the biomaterials grafted in the jaw must function at stress levels that are within the elastic region of their load/deformation curves, avoiding their permanent deformation, with the consequent failure of the implant above. In particular, the Printed@SmallPores samples showed also the highest values of compressive ultimate stress, probably due, as suggested before, to the thicker struts. On the other hand, the traditionally sintered samples showed a larger plastic region: This is also associated with an increased energy absorption and toughness of the biomaterial under compressive load up to fracture.

The SLA device used to produce the 3D-printed samples is equipped with a laser beam with a very small spot size; thus, it was expected to produce very detailed and highly defined structures, opening the possibility of customizing and varying the scaffold structure design. This fact is an innovative aspect in 3D printing and deserved a focused investigation, as here performed by means of SEM observations, which completed the integrated analysis of the BSBs.

By SEM, we observed that while the fracture in the SLA-3D-printed samples was localized in the structure nodes (Figure 5b,c) or on the external shells of the rods (Figure 4c,d), all the traditionally sintered samples revealed a ductile fracture surface (one example is Figure 6b). These observations show that, even on a surface level, the SLA-3D-printing production technique is very promising in order to offer effective anchorage surfaces for cell seeding and culture, which will be carried out at a later stage than this study.

Specifically, it could be argued that the sample size is too reduced to support the previous discussion of the results. Nevertheless, it has to be stressed that in our study, making use of microCT, we got significant morphometric results also on this narrow statistical sample, practically making no longer necessary the calculation of the statistical power, as previously discussed [40]. Indeed, the microCT data analysis is able to detect also minor deviations between samples, deriving from the stacking of 1000 successive 2D sections, each with a thickness of about 9.5 μm, and mapping the entire samples.

Moreover, as reported in the table in Figure 3, the standard deviations of the ultimate strength (MPa) and of the corresponding strain ε (%) between samples of the same group are low, allowing to produce reliable comparisons between test (SLA-printed) and control (traditionally sintered) samples.

At a general level, the SLA-3D-printed samples, due to the peculiar characteristics of the production technique, present a high level of accuracy, which is the sum of precision and trueness. Furthermore, for the sintered controls, being commercial products, reproducibility is guaranteed by the manufacturing companies that always guarantee also the size of the various products in their commercial forms, i.e., granules or, like in this study, small blocks.

## 4. Materials and Methods

Biphasic calcium phosphate (30% HA/70% β-TCP) samples, produced by SLA-3D-printing or by traditionally sintered methods, were tested basing on the experimental sequence that included the following actions:

MicroCT analyses of samples of each biomaterial: They served as control-references for the 3D morphometric analysis;

Load tests in continuous mode, with compression up to fracture: To reconstruct the stress/deformation curve in order to study the elastic characteristics and how they change between different biomaterials;

MicroCT of the same samples after the load tests: To achieve the 3D morphometric analysis for the prediction of the morphometric changes induced by compressive loading of the selected materials.

### 4.1. Biomaterials

The commercially available HA and β-TCP powders (Hitemco Medical Applications Inc, Old Bethpage, NY, US), which meet the specification of ASTM F1185-03 and ASTM F1088-04a, respectively, have been used. Fifteen g of 1-Morpholino-2-propen-1-one, 15 g of Propenoic acid, (octahydro-4,7-methano-1H-indene-5,?-diyl)bis(methylene) ester and 0.1 g of 2,4,6-trimethylbenzoyl-diphenyl-phosphine oxide were mixed, followed by stirring for 3 hours to prepare a photocurable resin. Afterwards, 21 g of HA powder and 49 g of β-TCP powder were added to the resin and stirred in the mixer for about 12 hours, in order to guarantee fluidity and homogeneity. Using a B type viscometer (“DV-E”, manufactured by Brookfield Engineering Laboratories, Inc, Middleboro, MA, USA), the viscosity of the obtained composition was measured at 25 °C, obtaining the result of 3400 mPa·s.

**Figure 7:**
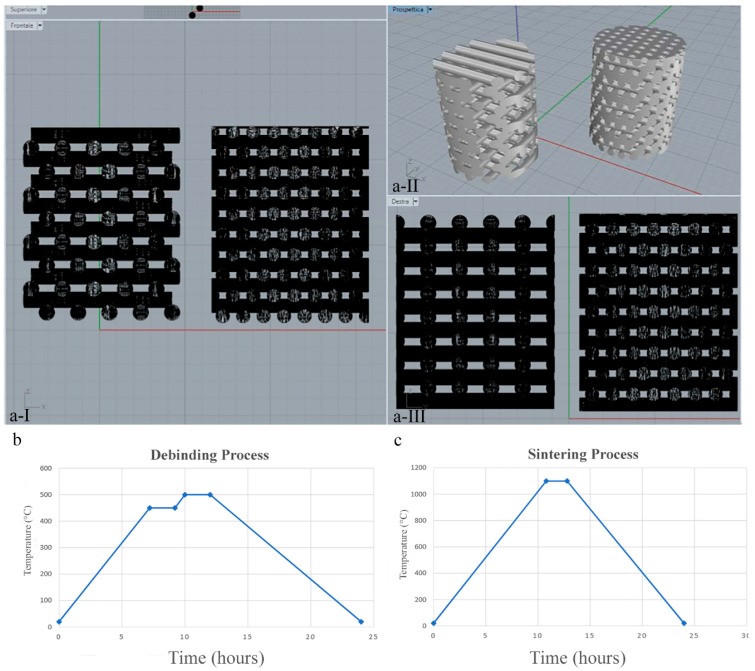
Three-dimensional printed samples design and production: (**a-I**) Printed@BigPores samples, with parallel links translated in the *x*-axis; (**a-II)** CAD reconstruction of the 3D-printed samples produced by laser light stereo-lithography (SLA); (**a-III**) Printed@SmallPores samples, with parallel links translated both in *x*- and in *y*-axes; (**b**) temperature vs. time graph of the debinding process; (**c**) temperature vs. time graph of the sintering process.

Two types of design have been studied as tests (Figure 7a):

Porous cylinders (nr. 2) with parallel links translated in the *x*-axis, hereinafter referred to as Printed@BigPores samples;

Porous cylinders (nr. 2) with parallel links translated both in *x*- and in *y*-axes, hereinafter referred to as Printed@SmallPores samples.

The cylinders were designed with the Rhinoceros® software (Mc Neel & Associates), obtaining a. stl file for each design. Subsequently, the files were processed with the Nauta® software (DWS, Thiene, Italy), creating appropriate media, and saved with the fictor extension, compatible with the chosen3D printer.

Using the previously described composition, 3D printing was performed by an SLA device in which light is irradiated from the bottom side through the light permeable bottom face of a shaping container, using a line drawing system (“DigitalWax 028J”, manufactured by DWS SRL, Thiene (VI), Italy ), under the following conditions: Laser output of 30 mW, wavelength of 405 nm, beam diameter of 0.02 mm, laser operation rate of 4600 mm/s, and single layer thickness of 0.06 mm in accordance with slice data based on three-dimensional CAD data.

Afterwards, the pieces were removed from the supports and washed in a beaker with ethyl alcohol placed in an ultrasonic bath for about 3 minutes. Subsequently, they were dried with compressed air to facilitate the release of residues of unpolymerized resin and alcohol from the pores.

The cylinders were then heat treated using the thermal cycle debinding process described in Figure 7b and then sintered with the thermal cycle described in Figure 7c.

Moreover, other biphasic calcium phosphate samples, with the same composition (30% HA/70% β-TCP), obtained by synthesis in the laboratory by the traditional way (Biotec srl, Povolaro, Italy), were tested to obtain control parameters: nr. 2 samples with applied sintering temperature of 1150 °C (Sintered@*T* = 1150 °C samples) and nr. 2 samples with applied sintering temperature of 1280 °C (Sintered@*T* = 1280 °C samples).

Finally, all the scaffolds were reduced to small blocks with a volume of about 1.4 cm³.

For comparative purposes, four healthy jawbones (nr. 2 from the maxillary and nr. 2 from the mandibular sites of different patients) have been also studied at morphometric level. Mean and standard deviation derived from these control jawbones was hereinafter indicated as Ctr@samples. These samples have been already investigated in previous studies for which the Ethics Committee approval was obtained (University of Varese, Italy; project identification code nr 826, 3^rd^ of October 2013). These studies protocols were explained to each involved subject, obtaining their signed informed consent. The samples were taken from patients undergoing traditional implant therapy. Instead of using a normal 2 mm diameter pilot drill, which would have milled the bone reducing it to a pulp, we used a core drill 2 mm in diameter to create the alveolus and obtain the studied bone cores.

### 4.2. High-Resolution Tomography (MicroCT)

A Skyscan 1174 tomograph (SkyScan-Bruker, Antwerp, Belgium) was set to a tension voltage of approximately 50 kV and a current of 800 μA. The tomographic imaging for this study was conducted at a resolution of 9.5 μm per pixel on a 180° rotation with a rotation step of 0.5° (9 s of exposure time per projection). The 1 mm filter of Al was used to adjust *x*-ray photon energy, attaining appropriate *x*-ray transmission through the samples. The total duration for tomographic imaging was approximately 1.5 h.

The absorption projection images (8 bit TIFF images) were reconstructed using the NRecon software (SkyScan-Bruker, Antwerp, Belgium) to obtain a set of cross-sectional slices (8 bit BMP images) of the columns, using ring artefact and beam hardening correction.

Three-dimensional images were generated using a Quad-Core Processor 2.01 GHz PC with 8 GB RAM and the commercial software VG Studio MAX 1.2 (Volume Graphics, Heidelberg, Germany). The image settings were based on the Scatter HQ algorithm with an oversampling factor of 5.0. *X*-ray contrast differences between the background and the biphasic calcium phosphate phase translated into two different peaks in the grey level scale. The Mixture Modeling algorithm (NIH ImageJ Plugin) was implemented to threshold the histograms. Thresholded slices were used to automatically separate the new biomaterial phase from the background.

The quantitative analysis was based on the structural indices usually measured for bone samples [41]: Sample surface to sample volume ratio (SS/SV—per millimeter), sample volume to total volume ratio (SV/TV—expressed as a percentage), mean strut thickness (S.Th.—expressed in micrometers) and mean strut thickness in the loading (compressive) direction (S.Th.Load.Dir—expressed in micrometers), mean strut number (S.Nr.—per millimeter), mean strut spacing (S.Sp.—expressed in micrometers) and mean strut spacing in the loading (compressive) direction (S.Sp.Load.Dir—expressed in micrometers).

### 4.3. Compressive Testing

The mechanical properties, when continuous compressive loading is applied up to fracture, were tested using a Material Testing Stage (MTS1) compatible with the Bruker SkyScan 1174 tomograph. It was easily installed in place of the standard sample holder; it is controlled using dedicated software, compatible with the main scanner control program. Indeed, with the 3D internal imaging offered by the microCT device, we had the unique opportunity to observe the results in situ. During scanning, the sample was subjected to a controlled compressive load and the loading curve was displayed on the screen in real time. An accurate force, simulating the compressive masticatory stress and measured by a load cell, was applied to the sample. A precision displacement sensor measured the resulting deformation.

The specifications of the MST1 stage are the following ones: Maximum force of 440 N, displacement sensor accuracy of ±0.01 mm, load measurement accuracy of ±4 N (±1% of the full range); maximum object diameter of 20 mm and maximum object height for compression of 23 mm.

### 4.4. Scanning Electron Microscopy

The Scanning Electron Microscopy (SEM) analysis of the biomaterials was carried out at the CISMIN Centre of the Polytechnic University of Marche. The samples, after compressive loading up to fracture, were mounted on appropriate stubs with conductive glue and gold-coated (Emitech K550, Emitech Ltd, Ashford, Kent, UK) before observation, at different magnifications, by a Philips XL20 (Philips Inc, Eindhoven, Netherlands) scanning electron microscope at 30 kV, equipped with an Edax microanalysis (EDS).

## 5. Conclusions

Strength in biomaterials is widely considered an umbrella term to describe their general integrity in terms of fracture resistance, quality and reduced fragility. To resist fracture, the biomaterial is expected to have a micro-morphometry that mimics the hosting bone site. Moreover, it should be as stiff as the hosting healthy bone to withstand deformation when loaded, but also be flexible to an extent and allow energy absorption during impact loading.

The biomaterials considered in this study were found to exhibit a morphometric structure similar to healthy jawbone, regardless of whether they were SLA-3D-printed or traditionally sintered at the different temperatures. However, fracture events in the SLA-3D-printed scaffolds were more similar to human jaw than in the sintered ones, as seen by observing the fracture surfaces by electron microscopy.

Moreover, the SLA-3D-printed samples (in particular, those with small pores and thick struts) showed the highest values of compressive ultimate strength, with smaller plastic region and toughness than the sintered biomaterials.

As previously shown by other authors [42], against a similar ultimate stress, the deformation achieved by the samples traditionally sintered at 1280 °C was significantly larger than in the samples sintered at 1150 °C. This could be due, at temperatures above 1150 °C, to allotropic transformation of TCP phase from β→α, where the α phase is reported to be a brittle phase, with reverse transformation not totally achieved during cooling [32].

This study was presented as the first step of a larger experimental project that will include, in short time, the study of the mechanical behavior of the SLA-printed scaffold under cyclic stress, simulating the chewing the jaw, and, as the next step, the analysis of cells adhesion to the biomaterial. In this context, it has to be underlined that the adhesion of cells to a biomaterial surface was found to be a major factor mediating bone regeneration. Indeed, several authors demonstrated and agreed that the micro-morphometry [35] and the micro-range surface roughness [43] modify, in a significant way, biocompatibility and tissue attachment in vivo. In particular, several researches have reported the enhancement of cell adhesion to biomaterials exhibiting an increased surface roughness [44,45], with a maximized number of contact zones, between cells and biomaterial, established through extending cytoplasmic processes and filopodia that enable the anchorage of the cells [46,47].

## Figures and Tables

**Table 1:** Morphometric analysis of microCT data, as measured in the different biomaterials before and after compressive tests. Bone control (ctr) parameters were obtained as mean between four different healthy jawbones, two in maxillary and two in mandibular human sites. Mean (SD); BCT: Before compressive test; ACT: After compressive test.

	Printed Big Pore BCT	Printed Big Pore ACT	Printed Small Pore BCT	Printed Small Pore ACT	Sintered *T* = 1150 °C BCT	Sintered *T* = 1150 °C ACT	Sintered *T* = 1280 °C BCT	Sintered *T* = 1280 °C ACT	Bone Ctr
SS/SV (mm^−1^)	5 (0)	7 (0)	4 (0)	5 (1)	17 (0)	17 (1)	19 (0)	20 (1)	18 (4)
SV/TV (%)	51.4 (0.3)	50.5 (0.3)	75.9 (4.7)	75.4 (2.4)	53.3 (0.1)	56.3 (0.6)	51.8 (1.1)	49.6 (0.3)	48.2 (8.5)
S.Th. (µm)	404 (11)	296 (1)	504 (82)	350 (61)	120 (0)	117 (7)	103 (6)	101 (6)	120 (23)
S.Th. Load dir (µm)	386 (40)	282 (20)	544 (49)	378 (81)	124 (0)	120 (4)	106 (6)	104 (6)	–
S.Nr. (mm^−1^)	1 (0)	2 (0)	1(1)	2 (1)	4 (0)	5 (0)	5 (0)	5 (0)	4 (1)
S.Sp. (µm)	380 (16)	290 (3)	157 (14)	116 (36)	104 (1)	90 (8)	96 (1)	103 (5)	130 (30)
S.Sp. load dir (µm)	364 (62)	277 (17)	172 (29)	145 (23)	107 (1)	93 (6)	99 (1)	106 (6)	–

## Abbreviations

BSBs	Bone substitute biomaterials
3D	Three-dimensions
SLA	Laser light stereo-lithography
MicroCT	Microtomography
